# The complete mitochondrial genome of the leafhopper *Idioscopus clypealis* (Hemiptera: Cicadellidae: Idiocerinae)

**DOI:** 10.1080/23802359.2017.1419083

**Published:** 2017-12-21

**Authors:** Dai Renhuai, Wang Jiajia, Yang Maofa

**Affiliations:** Institute of Entomology, The Provincial Key Laboratory for Agricultural Pest Management Mountainous Region, Guizhou University, Guiyang, P.R. China

**Keywords:** Mitogenome, Cicadellidae, *Idioscopus clypealis*, phylogeny

## Abstract

We have sequenced the complete mitochondrial genome (mitogenome) of the leafhopper *Idioscopus clypealis* in Idiocerinae, the mitochondrial genome sequences to be 15,393 bp with an A + T content of 78.3%. The mitogenome includes 13 protein-coding genes, two ribosomal RNA genes, 22 transfer RNA (tRNA) genes, and one non-coding [A + T]-rich region. These genes are in the same order as in the *Idioscopus nitidulus*. All protein-coding genes have ATN as the start codon, TAA and single T as the stop codon. The phylogenetic tree confirms that *I. clypealis* and *I. nitidulus* are clustered into a clade, and provides the relationships between the Megophthalminae, Deltocephalinae, Idiocerinae, Cicadellinae, and Typhloeybinae.

Leafhoppers belong to the family Cicadellidae, which is one of largest family in insects, about 22,000 species described worldwide (Camisão et al. 2014). The leafhopper *Idioscopus clypealis* is the pest of mango widespread species in the world (Fletcher and Dangerfield 2002; Khatri and Webb 2014). Previously, only nine complete mitochondrial genomes (mitogenome) of members of the Cicadellidae had been sequenced (Wu et al. 2016; Zhou et al. 2016; Wang et al. 2017; Yu et al. 2017). In this study, we have sequenced and annotated the complete mitogenome of *I. clypealis*, which is the second mitogenome in Idiocerinae. The specimen *of I. clypealis* used in this study was obtained from Yunnan Province, China in June 2016. The complete mitogenome of *I. clypealis* was sequenced using the next-generation sequencing method (Illumina HisSeq 2500 and 3Gb raw data, Berry Genomic, Beijing, China, more than 2 Gb clean data were acquired with each reads was 250 bp in length and the data quality was under standard of Q30 > 83%) and Sanger dideoxy sequencing. The voucher specimen’s genome DNA and male external genitalia are deposited in the Institute of Entomology, Guizhou University, Guizhou Province, China.

The complete mitogenome of *I. clypealis* is 15,393 bp in length (GenBank accession no. MF784430), containing 13 protein-coding genes (PCGs), 22 transfer RNA (tRNA) genes, two ribosomal RNA (rRNA) genes, and one non-coding [A + T]-rich region, while a repeat region is found in the [A + T]-rich region. Overall, the *I. clypealis* mitogenome has an A + T content of 78.3% (A 42.5%; T 35.8%; C 11.9%; G 9.8%), which is within the range reported for hemipteran mitogenomes (68.86–86.33%; Zhang et al. 2014). All PCGs have ATN as the start codon, TAA and a single T as the stop codon. All tRNA genes are identified by ARWEN version 1.2 software (Laslett and Canbäck 2008). The *16S* rRNA gene is 1198 bp in size and is located between *tRNA-L2* and *tRNA-V*; the *12S* rRNA gene is 747 bp in length and is located after *tRNA-V*. The control region is 1074 bp long, and is located between *12S* rRNA and *tRNA-I*, and there are 608 bp repeat regions in the non-coding region.

The phylogenetic relationships of the *I. clypealis* mitogenome assembly references and 11 other Hemiptera taxa from GenBank, were reconstructed using Bayesian analysis based upon the concatenated nucleotide sequences of the 13 PCGs (Figure 1). Sequences were aligned using MEGA6 software (Tamura et al. 2013) and poorly-aligned regions were trimmed for a final alignment of 9984 bp. Phylogenetic trees were generated using MrBayes (version 3. 2) software (Ronquist et al. 2011) with the TVM + I + G model. Bootstrap values obtained by 1,000,000 steps were indicated for each node. The phylogenetic tree confirms that *I. clypealis* is part of the Cicadellidae, all members of which are clustered into a clade. This provides the relationships between the Deltocephalinae, Megophthalminae, Idiocerinae, Cicadellinae, and Typhloeybinae.

**Figure 1.**
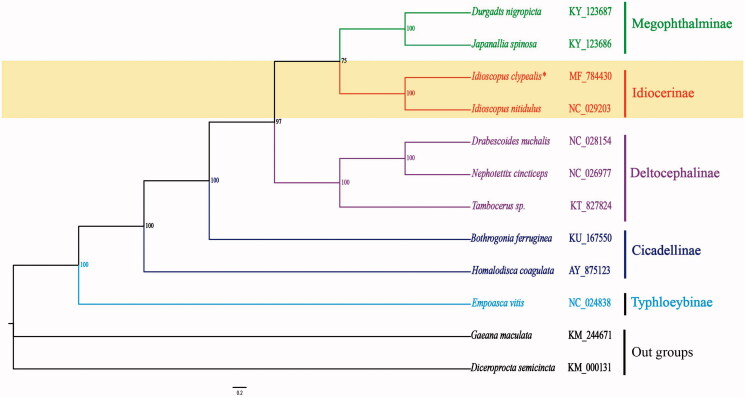
Phylogenetic relationships of the Cicadellidae based on the nucleotide sequences of the 13 concatenated PCGs, using closely related species of the Hemiptera.
